# Research on risk assessment model and simulation of online group polarization in emergencies

**DOI:** 10.1371/journal.pone.0305552

**Published:** 2024-06-17

**Authors:** Guoqiang Lu, Fang Liu

**Affiliations:** MuDanjiang Medicine University, MuDanjiang, HeiLongjiang, China; Rikkyo University, JAPAN

## Abstract

This study proposes a framework for the risk structure of group polarization in the online information of sudden public health incidents, as well as the causes and constraints of group polarization distributed in the ternary space. Then, combining the above two and based on the concept of phase change space, a simulation model of group polarization of online information in sudden public health incidents was constructed by reflecting the risk of group polarization with the number of network users who hold extreme views. The system dynamics simulation of the model is carried out by using the software Anylogic, and predict the evolution trend of the model from the perspective of simulation. The research results indicate that the model built in this study can effectively simulate the formation and dissemination of extreme opinions in the online public opinion of public health emergencies. The vital factors or constraints on the group polarization include emotional guidance, heat reduction, as well as and life pressure.

## 1. Introduction

Emergencies comprise natural disasters, accident disasters, public health events, as well as social security events. Over the past few years, a wide variety of emergencies have occurred frequently, causing great harm to people’s lives and property safety, and seriously affecting national security and social stability. The enthusiasm of users to express their opinions and attitudes in social media is increasing. The emergence of emergencies tends to cause the participation and discussion of network users, such that the network public opinion of emergencies is formed. The phenomenon of group polarization (GP) is one of the results of the evolution of public opinion. GP occurs when group members end up being more extreme in their position on a given issue after participating or being exposed to a discussion [1]. The influential work of Cass Sunstein identified in online information cocoons created by like-minded individuals [2–4], i.e., a key mechanism of GP, and hypothesized that GP will impoverish public discourse and favor extremism [5]. How to effectively control the group polarization in emergencies is considered a vital topic for the management of online information disorder and public attitude guidance in emergency crises.

With the development of mobile Internet technology and network social media platform, increasing GP phenomena are generated by network users for network public opinion events. Scholars have investigated GP identification and risk assessment of network public opinion in depth. Besides the "information cocoons" determined by the propagation dynamics, the "filtering bubble" arising from the recommendation algorithm is also one of the driving forces of GP [6,7]. The social comparison theory and the argumentation theory explain polarization as the output of the process, through which individuals reinforce their sense of membership to a group. Self-Categorization considers both theories and explains group polarization. Self-Categorization is a key mechanism through which individuals draw boundaries and make personal identity close to group identity based on an "Intra group and inter group" rhetoric [8,9], stereotypical communication [10]. The social antecedents of the formation of GP involve factors for communication dynamics based on individual psychological characteristics, as well as factors for external promotion (e.g., polarized media & opinion leaders, trolls, divisive events, as well as context). Homophily interaction in GP can be further facilitated or magnified by algorithms [11] and homogenous chains [12]. Existing research on Facebook [13] has suggested that the "disagreeable others" via Facebook news-feed boosts the growth of affective polarization. Numerous methods have been adopted to identify GP. Social network metrics typically assess social fragmentation among users [14,15] to detect biased interactions by tracking "friendship" relations [16,17] or conversational exchanges (e.g., retweets and mentions) [18]. Subsequently, content-based metrics based on a semi or fully automated text research have been proposed to identify ideologically separate communities [19], antagonist narratives[20,21], and the emergence of extreme beliefs [22,23], or polarized online sentiment [24].

In general, relevant research has placed a focus on the endogenous mechanism of GP, the factors, and the optimization of GP identification methods. However, there have been rare research results on GP risk assessment from a broader perspective under the background of emergency crises. The GP control strategy is introduced in the diffusion of extreme opinions, and there has been scarce dynamic analysis of the GP control process. Considering the effect of crisis factors in emergency situations on the formation of GP, this study builds a risk assessment model of GP while conducting global dynamic simulation on the GP formation, so as to achieve the suppression of GP.

The following three problems are primarily solved in this study: ①Combined with the formation mechanism analysis of GP in the framework of ternary space analysis, the risk assessment model of GP of online information in emergencies is built; ②With the "Pfizer COVID-19 small-molecule drug" incident as an example, the scale and inhibitory factors of negative emotions and extreme opinions of online users were determined; ③The specific strategies for guiding and controlling the GP are proposed through sensitivity analysis of the parameters of the GP risk assessment model.

## 2. Literature review

### 2.1 Ternary space

Ternary space comprises physical space, social space, and information space as relatively independent cohesive modules. The above modules are coupled and blended with each other to establish an organic whole. The information & physical’ fusion system should be listed as a vital research project in the American competitiveness plan, as initially suggested by the American Academy of Sciences (NAS) in 2006. The above system primarily aims to achieve the organic integration and deep cooperation of computing, communication and control technologies and physical systems based on the binary integration of information space and physical space [25]. In 2010, the research conducted by the Chinese Academy of Sciences has suggested that, the physical-information system (cyber physical system), as driven by big data, will develops into a three-dimensional space [26]. In the same year, Guojie Li, an academician of the Chinese Academy of Sciences, highlighted that the current world of information is a dynamic and open network society that comprises a considerable number of people, machines and objects, i.e., a ternary space that covers of physical space, information space, and human space [27]. In 2018, Weicheng Fan, an academician of the Chinese Academy of engineering, suggested that the large-scale emergency cloud service system is completed in the framework of ternary space [28]. The research from Tsinghua University in 2021 conducted in-depth modeling on the association representation of ternary spatial heterogeneous data in accordance with the topological graph theory, thus expanding the mathematical calculation boundary of ternary space data mapping [29]. The ternary space theory, following the physical world and information space, considers the social and human factors in the real world while integrating physical space, information space, and social space to build a cross domain system. The application research of the ternary space theory aims to map the contents of physical space and social space to information space through cooperative perception, integrate and analyze information coupling effect in information space, while exploring the retrospective guidance path to physical space and social space.

### 2.2 GP of online information

The phenomenon of GP originates from the irrational characteristics of the masses. Scholars in the fields of philosophy, economics, politics, and social psychology all proposed the irrational characteristics of the masses and studied the formation mechanism of the phenomenon of GP. The research on the phenomenon of GP was initiated in the 1960s, and the relevant theories have relatively matured. In 1961, James Stoner’s comparison between individuals and groups on risky decision-making was considered the starting point of the research on GP [30]. Sunstein initially studied GP from the perspective of the Internet and highlighted that GP also exists in the online social networks. In addition, online GP refers to a mapping of social polarization [2]. Since public opinion events generate issues driven in social media for network users, the interaction behavior of network users facilitates the aggregation of opinions while expediting the formation of online GP [31]. The method of controlling experiments that was proposed by Michael Wallach and Nathan Kogan has confirmed group discussion as a sufficient and necessary condition to generate GP [32]. The choice conflict theory in psychology complements the social norms theory in explaining the phenomenon of GP. And the social identity theory can explain the initiative of group members more specifically [33]. When individuals face multiple options, the chances of postponing decisions, seeking alternatives and choosing default options increase significantly [34]. Existing research has confirmed that when group members participate in discussions, they often choose irrational solutions against the principle of rationality when facing difficulties in decision-making, such that GP is formed.

### 2.3 Theoretical model of GP

Numerous theoretical models applying to the study on GP have been built based on the social norms theory, the strong argument theory, and the self-classification theory, combined with opinion dynamics. The agent-based computational modelling framework has aroused attention from scholars over the past few years. Some [35,36] have developed agent-based models of GP based on classic bounded confidence models [37,38]. Others have proposed models generating opinion polarization due to agents’ biased-assimilation to social effect [39]. Two further prominent classes of models on opinion polarization refer to the models of negative effect [40,41] and those of social effect based on persuasive arguments [42], both of which root in classic sociological and psychological theories of polarization. The risk of GP poses a serious hazard to society, and the group scale in GP is generally large, such that research is difficult to conduct experimental methods in practice. Accordingly, the simulation method can be a better choice for the research on GP. Although the simulation data is not real data, the simulation model is capable of more effectively indicating the role played by the GP factors. For instance, simulation methods have been employed by existing research to study the correlation between group separation and opinion polarization [43]. The existing theoretical models have often stressed one aspect of the formation process of GP, which is either the opinion dynamics or the external motive force, as well as lack of systematization and comprehensiveness. This study, based on the ternary space, involves the causes of GP as comprehensively as possible to promote the fidelity and extrapolation of simulation.

## 3. Risk assessment model of online GP based on ternary space

### 3.1 Transmission mode of GP risk of emergency in ternary space

According to the definition of GP, the core elements involve irrational interaction and extreme opinions. Irrational interaction has been confirmed as a necessary condition for GP, and extreme opinions are the result of GP. The GP risk of online information in emergencies is a complex system, which occurs and forms under the coupling and superposition of a wide variety of risk elements in the ternary space.

**Fig 1:**
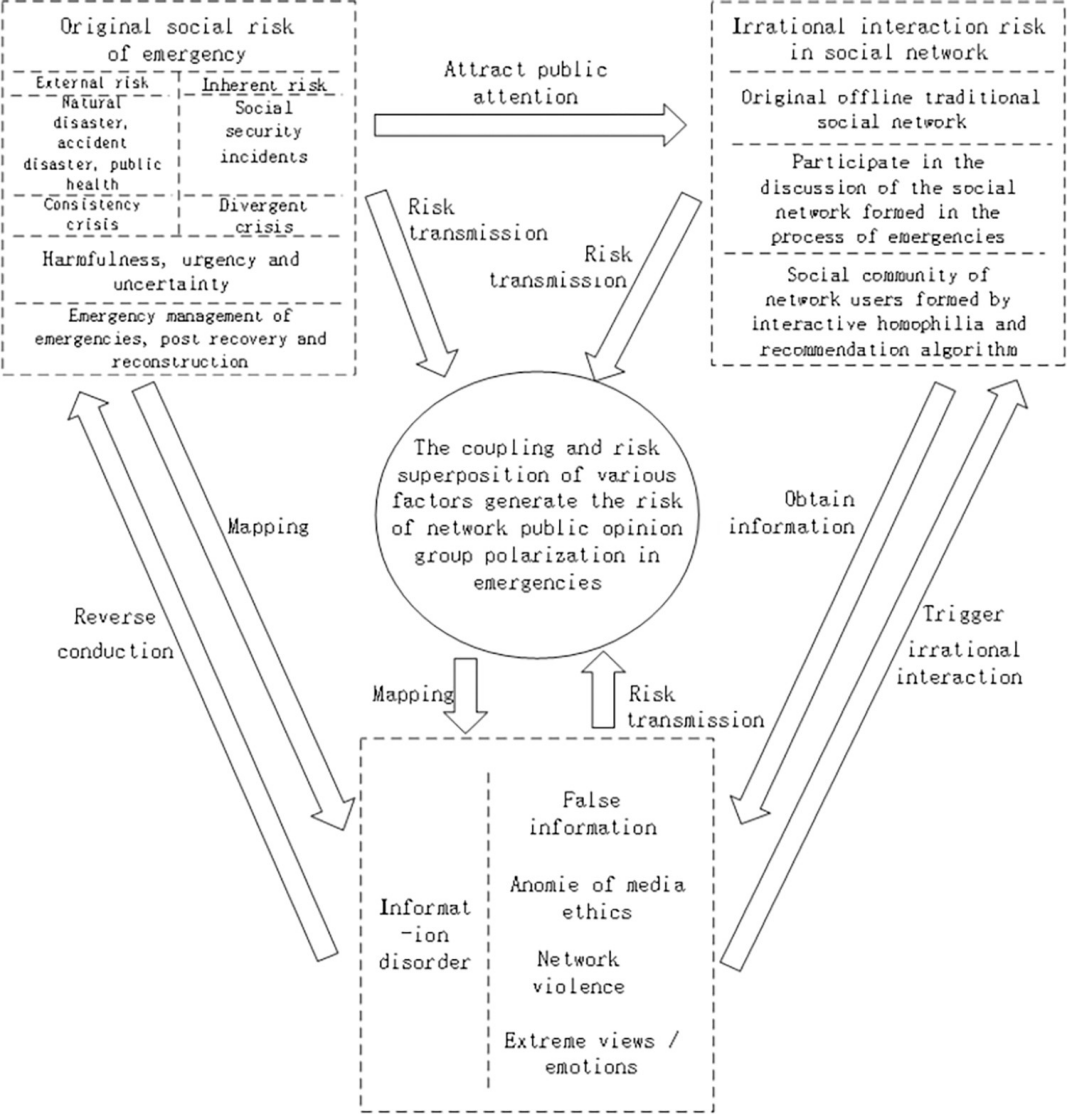
The structural framework of the GP risk of network public opinion in emergencies.

To gain more insights into the distribution of GP risk of online information in emergencies in the ternary space and the transmission of risk structures in different spaces, a GP risk structure framework integrating risk elements, risk performance in emergency situations and risk transmission paths is built to elucidate the coupling of risk elements, risk superposition and risk transmission. Fig 1 illustrates the structural framework of the GP risk of online information in emergencies.

The framework of GP risk structure of online information in emergencies comprises the original social risk of emergencies in physical space, the irrational interaction risk of users in social space, as well as the information disorder risk in information space. The original social risks of emergencies include external risks and internal risks. In accordance with Anthony Giddens’ understanding, natural disasters, accident disasters and public health events basically fall into the scope of "external risks", while social security events belong to "internal risks". External risks are usually less difficult to deal with than internal risks. According to the understanding of Enricol Quarantelli, the first three categories basically belong to "consistency crisis", and social security events belong to "divergence crisis", which often results in group events. Natural disasters, accident disasters and public health events mainly cause casualties, property losses and other social hazards; Social security events usually point the spearhead directly at the core system, mainstream values, or social structure, challenging the legitimacy of the existing order. In emergencies, the original social risks caused by the harm, urgency and uncertainty of the events attract the attention of many people, and map to information space through information dissemination. The new social relationship networks formed by the people in social space, and the discussion are also mapped to information space. Network users carry out irrational interaction behaviors when driven by the disorder information in information space. They build a more closely related social relationship network when driven by interaction affinity and recommendation algorithms, increasing the risk of users’ irrational interaction. Thus, the risks from physical space and social space are integrated as the information disorder risk in information space under the management department’s response error and the media’s anomie of information dissemination. The GP risk is formed under the coupling effect of a wide variety of risk elements in the ternary space, resulting in group extreme opinions. It exists as big data of risky user preference in information space, affects the decision-making and management of emergencies, has a certain effect on disaster recovery and reconstruction.

### 3.2 GP risk assessment model of online information in emergencies

**Fig 2:**
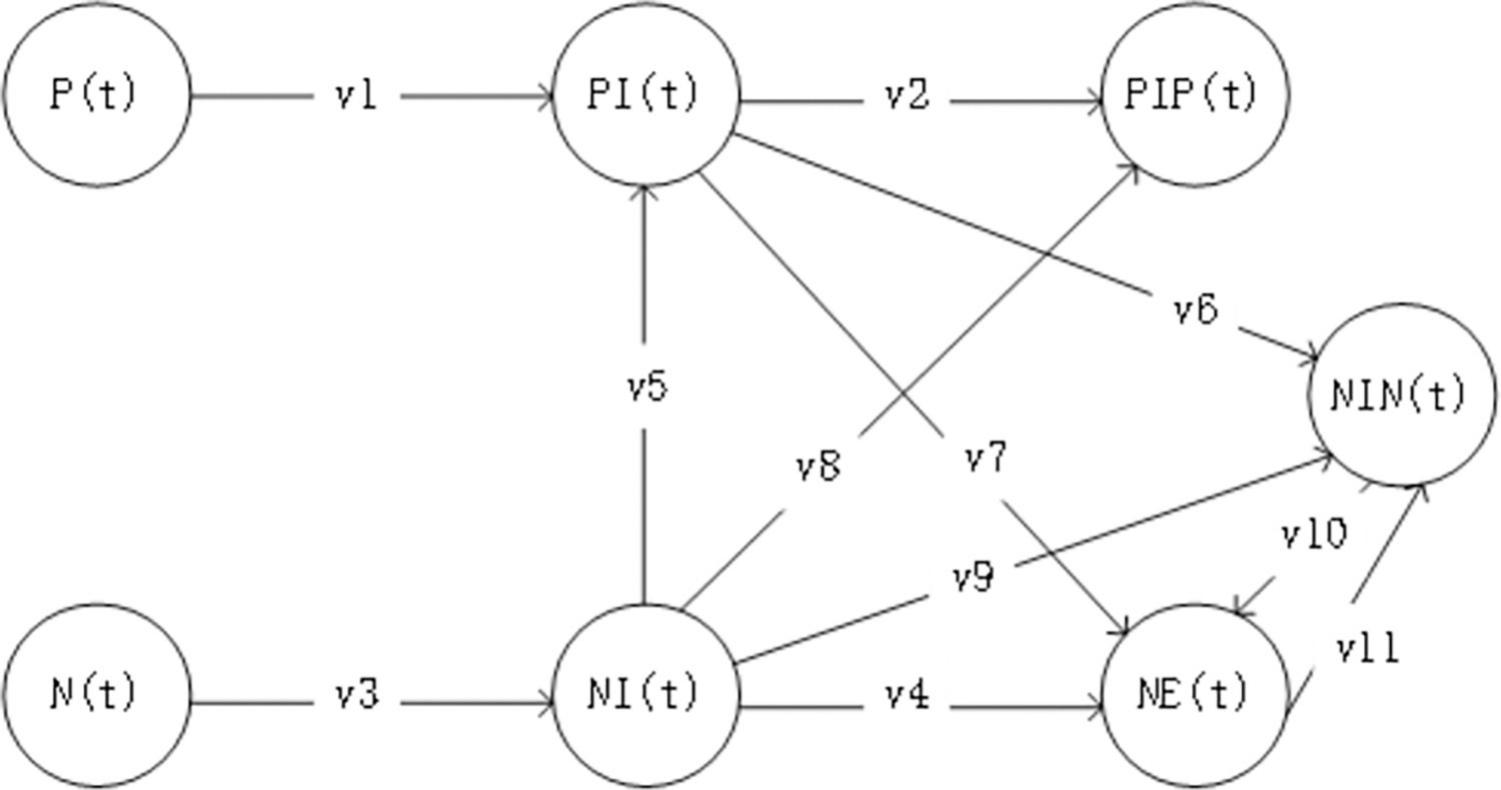
Conceptual model of GP risk assessment for emergencies.

In 2006, Grabowski and others proposed that based on Ising model, they first realized the interpretation of social public opinion through computer modeling and simulation [44]. Since then, the idea of using computer modeling and simulation to solve social public opinion problems has become popular. The research on GP has also made a breakthrough. The application of public opinion evolution models such as Sznajd model, voter model, Weisbuch-Deffuant (WD) model and Hegselmann-Krause (HK) model has made great progress in the field of GP simulation research. According to the transmission mode of GP in the ternary space, the risk factors of GP are deconstructed in the ternary space. Under the effect of a wide variety of causal factors, there are seven states of network users, including users with initial positive opinions and do not engage in the social network P(t), i.e., users with positive opinions at the beginning of public opinion focusing on emergencies; Have initial positive opinions and conduct interaction PI(t), i.e., although some users have positive opinions after the public opinion on emergencies, not all users participate in the discussion, and only some users participate in the discussion and interact with other users; The user has a positive opinions PIP(t) after interaction, i.e., the state of PIP(t) is that they (PI (t) or NI (t)) continuously hold positive views after interaction, and exit the public opinion field as the information dissemination lifecycle ends.; Having an initial negative opinions and do not engage in the social network N(t), i.e. users who have negative opinions at the beginning of public opinion on emergencies; Have initial negative opinions and interact NI(t), i.e., although some users have negative opinions after the public opinion on emergencies, not all users participate in the discussion, and only some users participate in the discussion and interact with other users; With negative extreme opinions NE(t), i.e., with the development of events and the continuous interaction of network users, some users have formed extreme opinions with negative emotions; With negative non-extreme opinions NIN(t), i.e., with the development of events and the continuous development of network user interaction, some users have formed negative but not extreme opinions. The conceptual model(The first abstraction from the real world to the information world) of GP risk assessment for emergencies is shown in Fig 2. The relevant indicators involved in the GP risk assessment model are explained in Table 1.

**Table 1:** Interpretation of relevant indicators of GP risk assessment model of network public opinion in emergencies.

Parameter	Parameter meaning	Parameter	Parameter meaning
**t**	At a certain time in the process of network public opinion propagation of emergencies t	***V6***	Change rate of users holding positive opinions to users holding negative non-extreme opinions
***P(t)***	At a certain time t, the number of users with positive opinions at the beginning of network public opinion focusing on emergencies	***V7***	Change rate of users holding positive opinions to those holding negative extreme opinions
***PI(t)***	The number of users who in P(t) interact with other users at a certain time t	***V8***	Change rate of users with negative opinions changing to positive opinions after participating in the discussion
***PIP(t)***	At a certain time t, the number of users with positive opinions after interacting with other users	***V9***	Change rate of users holding negative opinions to users holding negative non-extreme opinions
***N(t)***	At a certain time t, the number of users with negative opinions at the beginning of network public opinion focusing on emergencies	***V10***	Change rate of users from negative non-extreme opinions to negative extreme opinions
***NI(t)***	Number of users who in N(t) interact with other users at a certain time t	***V11***	Change rate of users from negative extreme opinions to negative non-extreme opinions
***NE(t)***	At a certain time t, the number of users with extreme opinions with negative emotions	***λ***_***1***_	Government response impact
***NIN(t)***	At a certain time t, the number of users with negative emotions and non-extreme opinions after interacting with other users	***λ***_***2***_	Effect of emotional guidance
***V1***	Change rate of user participation in discussions with positive opinions	***λ***_***3***_	Effect of heat reduction
***V2***	Users having positive opinions maintain the change rate of positive opinions after participating in the discussion	***λ***_***4***_	Coupling effect of other events
***V3***	Change rate of users with negative opinions participating in discussions	***λ***_***5***_	User risk propensity
**V4**	Users with positive opinions will generate negative extreme opinions after participating in the discussion	***λ***_***6***_	Information uncertainty
**V5**	Change rate of users holding negative opinions to users holding positive opinions	-	

As depicted in Table 1, the risk assessment model of GP in emergencies built in this study fully considers the causes or constraints of GP in the ternary space. Unexpected events in physical space are the source of network public opinion. The model built in this study covers the effect of government response in the sudden crisis in physical space*λ*_*1*_, effect of emotional guidance*λ*_*2*_, effect of heat reduction*λ*_*3*_, Coupling effect of other events*λ*_*4*_; In social space including user risk propensity*λ*_*5*_; In information space including information uncertainty*λ*_*6*_ and change rate of network user status based on social identity theory. That is, the effect of the ratio of positive emotion to negative emotion on the change of users’ opinions.

First, systematicness and comprehensiveness are important requirements for social risk assessment and important guarantee for the accuracy. The GP risk assessment model based on the GP risk transmission mode of emergencies in the ternary space fully indicates the mapping of the contents in physical space and social space to information space through collaborative perception, the information integration and analysis under the coupling effect of the ternary space in information space, and the reverse guidance path of physical space and social space to information space. Second, the model built in this study fully considers the dynamic mechanism of Internet public opinion transmission. According to the social comparison theory, GP is the result of people’s desire to be accepted and understood by the group. To achieve the goal of being accepted and understood by the group, individuals must constantly observe the social expression of other people, and continuously modify and even reshape their self-awareness and self-expression according to the norms recognized by the group and society, and finally form a similar but slightly extreme attitude and position with others [45]. Thus, the effect of the ratio of users with positive and negative emotions is set when the state of network users is changed in the model, i.e., network users will observe the situation in the public opinion field in advance and are inclined to select the opinions with a larger number of users when entering the public opinion field. Third, the time of implementing the public opinion guidance and control strategy in the model is consistent with the characteristics of emergencies and the reality of online information guidance. After an emergency occurs, considerable negative emotions are generated in the network public opinion field due to the uncertainty and hazard of the emergency. In practice, the management department has usually conducted emotional guidance using the methods of information disclosure and agenda setting. Once the extreme opinions (i.e., an essential symbol of GP) are established, the management department, considering the hazard posed by the extreme opinions representing the irrational interaction of network users, often adopts the more direct public opinion guidance and control strategy based on the restricted traffic. Accordingly, the model in this study makes emotional guidance play a role in the user interaction stage, and when extreme opinions are formed, it makes the effect of heat reduction play a role. To sum up, the GP risk assessment model of online information in emergencies built in this study considers the comprehensiveness of risk factors in social risk assessment, the dynamic mechanism of network public opinion propagation in emergencies, and the actual implementation time of public opinion guidance and control, ensures the accuracy of GP risk assessment.

### 3.3 Parameters of simulation model for GP risk assessment

There are numerous causes and constraints for the polarization risk of online information in emergencies. The above relevant factors exist in the ternary space, and together act on the network users to promote them to change among the seven states. The effect of government response, including timeliness and rationality, is the specific measures taken by government in emergency management. The timeliness of response can indicate whether the government makes a rapid response after the occurrence of an emergency. So is the rationality of government response, play an important role in emergency management. Emotional guidance represents the management department’s efforts to achieve the goal of transferring negative emotions through active publicity and reporting in response to the negative emotions existing in the public opinion field after the occurrence of an emergency. The degree of heat reduction indicates the measures taken by the government to limit the data traffic when the information disorder occurs. Some research from the perspective of public opinion transmission mechanism has confirmed that reducing data traffic and suppressing heat is effective to information disorder. The above three factors are the conditions for physical space to suppress GP. In accordance with the archetypal concept in self-categorization theory [46], "stereotype" largely accounts for the formation of GP. Besides, "stereotype" must originate from the effect of other events on users’ cognition. Thus, it can be considered that "coupling with other events" is the driving force of GP. In social space, the social relationship network is mainly embodied as an online social relationship network that comprises reading, forwarding, comments, likes and other interactive behaviors. Due to the anonymity of social media platforms and other characteristics, online users are more likely to have negative emotions or extreme opinions as compared with offline users. The greater the risk propensity of individual, the easier it will be to form extreme opinions. Uncertainty has been confirmed as the main feature of emergencies. Social media platforms map the uncertainty of emergencies in physical space to information space, forming a large amount of uncertain information about emergencies, even including false information, malicious information, and error information. The causative and restrictive factors of GP in the ternary space work together and play different roles at different stages of the development of public opinion.

### 3.4 Simulation model and rules for GP risk assessment

Due to suddenness, uncertainty and hazard, emergencies arouses the attention of considerable network users and allows them to engage in the discussion. In that case, the government should deal with the effect *λ*_*1*_, the network users become the initial users of the public opinion in emergencies at a certain rate. The greater the effect of government response(*λ*_*1*_), the smaller the number of initial users. The initial user delays entering the public opinion field by a certain proportion. After the initial users enter the public opinion field, they would immediately differentiate from the temporary state of no opinion into the users with positive or negative opinions. On that basis, the number of differentiation ratios was affected by the risk propensity (*λ*_*5*_) of individual. Besides, the social comparison theory suggests that the proportion of existing users with positive or negative opinions affect the opinion choice of users who delay entering into the online public opinion field. Eq (1) expresses the number *S*_*n*_ of users forming negative opinions.


$$S_{n}=None*\lambda_{5}//*(P(t)*N(t)>0?N(t)/(P(t)+N(t)):0.5)$$ Eq (1)

In Eq (1), "*None*" denotes the number of users whose specific opinions are unformed. The choice of opinion users is suggested determined by the ratio of risk prone users, i.e., the number of negative opinion users. Users without any opinions for the time being would observe the number of users with positive or negative opinions in the public opinion field before developing their opinions. When the number of users having positive or negative opinions reaches 0 (P(t)*N(t) = 0), a negative opinion is formed with a probability of 0.5. When P(t)*N(t) > 0, first divide *N(t)*/*P(t)*, then a negative opinion is formed with the probability of *N(t)*/*P(t)*. "//" refers to integer division. Because the calculation is based on the number of people, divide the result by 1 to obtain the integer part of the value. Similarly, the number *S*_*p*_ of users forming a positive opinion is shown in Eq (2).


$$S_{p}=None*(1-\lambda_{5})//*(P(t)*N(t)>0?P(t)/(N(t)+P(t)):0.5)$$ Eq (2)

(1) Some of the network users who have formed positive opinions have not participated in the discussion and left the network public opinion field, while the other part is affected by information uncertainty *λ*_*6*_. The composition of the network users of the positive opinion at time t is shown in Eq (3).


$$\frac{dP(t)}{dt}=None*(1-\lambda_{5})//*(P(t)*N(t)>0?P(t)/(N(t)+P(t)):0.5)-P(t)*V1-P(t)*time()$$ Eq (3)

In Eq (3) *V1 = λ*_*6*_*/T*_*pi*_. *T*_*pi*_ is the duration of network user interaction with a positive opinion. The time() is a built-in function in the simulation system, which can obtain the current running time of the model.

On the one hand, the network users *PI(t)* with positive opinions interact with each other comes from *P(t)*. On the other hand, there are also network users with negative opinions who change from *NI(t)* after interactive discussion. Changing from *P(t)* to *PI(t)* is subjected to information uncertainty as described above *λ*_*6*_, changing from *NI(t)* to *PI(t)* is guided by emotion *λ*_*2*_, corresponding change rate V5 = NI(t)*λ_*2*_*/T*_*c*_. *T*_*c*_ is the emotion conversion time, i.e., the time required to change from positive emotion to negative emotion and vice versa. Moreover, some users in *PI(t)* continue to maintain positive opinions after interaction and finally leave the public opinion field, there is also a part that changes to the negative non-extreme opinion *NIN(t)* at the rate *V6*. *PI(t)* under “coupling with other events” *λ*_*4*_ to generate network users with “stereotyped prejudice” by randomly generating a true / false method and change to a negative extreme opinion *NE(t)* at a rate *V7*, *V7 = randomTrue(NIN(t)/NE(t)*?(PI(t)*_4_*)/(T*_c_*T_*e*_*)*:(PI(t)*NIN(t)/ NE(t))/(T_c_*T_*e*_*))*. *T*_*e*_ indicates the formation time of extreme opinions. Accordingly, the composition of *PI(t)* at time t is as shown in Eq (4).


$$\frac{dPI(t)}{dt}=PI(t)*\lambda_{6}/T_{pi}+NI(t)*\lambda_{2}/T_{c}-PI(t)/T_{c}-PI(t)*time()-PI(t)*V7$$ Eq (4)

Among the user states relating to the positive opinion, *PIP(t)* suggests that the user having a positive opinion continuously maintains the positive opinion after discussing and interacting with other network users. Except that *PI(t)* is converted into *NIN(t)* and *NE(t)*, other users having positive opinions leave the network public opinion field with the running time of the model.

(2) After the initial user forms a negative opinion (*N(t)*), some users choose to interact with other users to enter the *NI(t)* state, the other part directly exits the public opinion field, the transition process and equation are similar to the state corresponding to *P(t)* and will not be repeated here. The network users in *NI(t)* state, besides the above *λ*_*2*_ to *PI(t)* and leave the public opinion field, some users change to negative non-extreme opinion *NIN(t)* under the effect of *λ*_*2*_ at the rate *V9*, *V9* = NI(t)*λ_*2*_/*T*_*ef*_, *T*_*ef*_ is the extinction time of extreme opinions; A part of the users change to the negative extreme opinion *NE(t)* at the rate *V4*, *V4* = *NI(t)*/*T*_*e*_. The composition of *NI(t)* at time t is shown in Eq (5).


$$\frac{dNI(t)}{dt}=N(t)*\lambda_{6}/T_{ni}-NI(t)/T_{e}-NI(t)*time()-NI(t)*\lambda_{2}/T_{c}-NI(t)*\lambda_{2}/T_{ef}$$ Eq (5)

In Eq (5), *T*_*ni*_ is the interaction time of network users having a negative opinion. According to the definition of GP, when users with negative opinions continue to interact without the effect of external factors, based on the interpretation of self-categorization theory and social comparison theory, extreme opinions with negative emotions gradually form, thus evolving into GP phenomenon. However, in this process, not all network users have formed extreme opinions, and some users have changed to users with negative non-extreme opinions. Some users in the *NIN(t)* exit the public opinion field, some users with “stereotyped prejudice” *λ*_*4*_ affects users who transition to *NE(t)* with a negative extreme opinion at a rate *V10*, *V10* = NIN(t)*λ_*4*_/*T*_e_*NIN(t)/*NE(t)*. The composition of *NIN(t)* at time t is shown in Eq (6).


$$\frac{dNIN(t)}{dt}=NE(t)*\lambda_{3}/T_{ef}+NI(t)*\lambda_{2}/T_{ef}+PI(t)/T_{c}-NIN(t)*\lambda_{4}/T_{e}*NIN(t)/NE(t)-NIN(t)*time()$$ Eq (6)

In Eq (6), the first item is that when extreme opinions appear in the public opinion field, the management department takes more direct intervention measures, i.e., reducing the popularity of relevant online information by restricting traffic, banning words, and adding other topic settings, so as to achieve the purpose of controlling extreme opinions. The specific explanation is: Network users with extreme opinions *NE(t)* under the effect of*λ*_*3*_ at the rate *V11* = NE(t)*λ_*3*_/*T*_*ef*_ change to *NIN(t)*. In accordance with the self-categorization theory, extreme opinions are generally archetypal, i.e., rigid, and less likely to change from extreme opinions to positive opinions [46]. On that basis, *NE(t)* in the GP risk assessment simulation model for emergencies built in this study only has the flow that changes to *NIN(t)* and exits the public opinion field state, whereas the flow that *NE(t)* changes to *PI(t)* is not set. The composition of *NE(t)* is expressed in Eq (7).


$$\frac{dNE(t)}{dt}=NIN(t)*\lambda_{4}/T_{e}*NIN(t)/NE(t)+PI(t)*V7+NI(t)/T_{e}-NE(t)*\lambda_{3}/T_{ef}-NE(t)*time()/T_{ef}$$ Eq (7)

The network user *NE(t)* forming an extreme opinion is the user state that the GP risk assessment model focuses on. Because according to the definition of GP, the core elements of GP include irrational interaction and extreme opinions. Irrational interaction is a necessary condition for GP, and extreme opinions are the result of GP. The above equation are the expression of the dynamic state of the 7 groups of people in the network public opinion GP risk assessment model for emergencies.

## 4 Simulation analysis and data results

### 4.1 Case selection of network public opinion GP in emergencies

**Fig 3:**
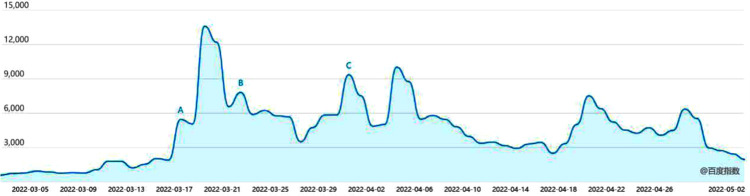
"Pfizer coronavirus small-molecule drug" heat trend chart (from "Baidu Index").

At the end of 2019, COVID-19 broke out worldwide. As of May 2022, there were 511479320 confirmed cases of COVID-19 and 6238832 deaths in the world. On November 5, 2021, Pfizer biopharmaceutical company of the United States announced that its COVID-19 oral drug could reduce the risk of hospitalization and death of high-risk COVID-19 patients by 89%. On November 30, 2021, the US Food and Drug Administration (FDA) approved the emergency authorization application of Pfizer’s new coronavirus treatment drug Paxlovid, and stated that Paxlovid was used to treat non hospitalized adults with high risk of developing severe diseases. On February 11, 2022, China’s State Food and Drug Administration conditionally approved Pfizer Paxlovid to be listed. On March 15, Pfizer Paxlovid was included in the latest diagnosis and treatment plan. On March 17, the first batch of 21200 boxes arrived in Shanghai and were distributed to many provinces. On March 21, Pfizer Paxlovid was included in the scope of medical insurance payment by the National Medical Insurance Bureau. On March 27, the price of Paxlovid was quoted as ¥2300 per box, and one box was a course of treatment (5 days). On April 16, the media reported that China’s Shanghai Pharmaceutical Group cooperated with Pfizer. The "Pfizer COVID-19 small-molecule drug" incident is one of the many hot events in the development of COVID-19 in 2022, attracting the attention and participation of numerous Internet users. In the process of network user interaction, due to the use of medical terminology in relevant news reports, the lack of medical knowledge of some network users, and the lack of familiarity with the field of new drug research and development, network users have irrational interaction, resulting in extreme opinions such as using imported western medicine as unpatriotic and forming GP risk. The heat trend of "Pfizer coronavirus small-molecule drug" is shown in Fig 3 (From “Baidu Index”).

### 4.2 System dynamics model

**Fig 4:**
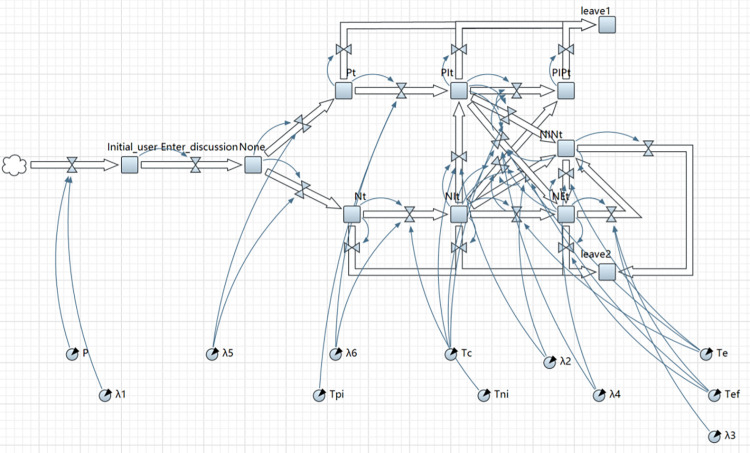
System dynamics model of GP risk of network public opinion in emergencies.

According to the real background of the "Pfizer COVID-19 small-molecule drug" incident, the system dynamics method is adopted based on the above-mentioned public opinion group polarization risk assessment model to design the system dynamics model using the Anylogic software. Fig 4 illustrates the model implementation.

Fig 4 illustrates the transition paths between different states of network users and the causes or constraints distributed in the ternary space during the formation of polarization GP risk in emergencies. Positive or negative emotions are often generated in the public opinion field since there are a series of complex factors (e.g., disaster causing subjects, response measures and information disclosure) with the occurrence and development of emergencies. However, Internet users with positive or negative opinions choose to continue to pay attention and participate in the discussion under the stimulation of the social harm and uncertainty that are common in emergencies. A large-scale interactive discussion of network users for emergencies constitutes a necessary condition for GP on the social media platform. Under the sudden crisis, if the network users have stereotyped prejudice, they will evolve to extreme opinions. In this process, the emotional guidance taken by the government and the reduction of flow and heat will restrict the formation of GP, and even make the formed extreme opinions gradually reverse into negative non-extreme opinions or positive opinions. Lastly, network users in a wide variety of states will exit the public opinion field, which complies with the law of the life cycle of network public opinion information dissemination. The end of information dissemination does not indicate that the risk of GP is negligible since the GP of information space in the ternary space is most likely to fed back to physical space and hinders the emergency management of emergencies, thus reducing the resilience of the city in crises.

### 4.3 Simulation results of system dynamics model

The public opinion development trend of the "Pfizer COVID-19 small-molecule drug" incident suggests that relevant reports appeared in February, whereas this incident had aroused a relatively low attention from Internet users. At the beginning of March, the popularity of relevant Internet public opinion began to rise. However, there was no new report relating to the "Pfizer COVID-19 small-molecule drug" incident in early March. Thus, it is preliminary judged that this incident was coupled with the COVID-19 outbreak in some areas of China in early March. The new outbreak raised Internet users’ concern about the relevant drugs and treatment plans. Adjust the model parameter values with the goal of simulating the real process of Pfizer events. In another paper, the author of this paper has completed calculations based on the meta contrast ratio for the quantitative identification of GP in the Pfizer event [47]. The parameter values of the network public opinion GP simulation model for emergencies are set (Table 2).

**Table 2:** Parameter setting of GP simulation model of network public opinion in emergencies.

parameter	Value meaning of parameter value setting	Parameter value
**S(0)**	Number of initial users in the emergency network public opinion field	100 people
***λ***_***1***_	The effect of government response measures on the number of users who pay attention to Internet public opinion events under emergency crises	0.2
***λ***_***2***_	The effect of the emotional guidance measures taken by the management department against the negative opinions in the public opinion field on the attitude change of network users	0.2
***λ***_***3***_	The effect of traffic restriction measures taken by the management department against extreme opinions in the public opinion field on the attitude change of network users	0.4
***λ***_***4***_	Under the sudden crisis, coupling with other events, the network users have a negative impact on the users or changes to a negative opinion	0.9
***λ***_***5***_	The proportion of Internet users with negative emotion tendency due to their individual personality characteristics	0.2
***λ***_***6***_	The effect of network users’ interactive discussions with other users due to the uncertainty of information of unexpected events	0.5
***T***_***c***_	Time required for positive and negative opinions to change each other	2 days
***T***_***e***_	Time required to form extreme opinions	6 days
***T***_***ef***_	Time required for extreme opinions to subside	7 days
***T***_***pi***_	Interaction time between network users with positive opinions and other users	3 days
***T***_***ni***_	Interaction time between network users with negative opinions and other users	1 days

**Fig 5:**
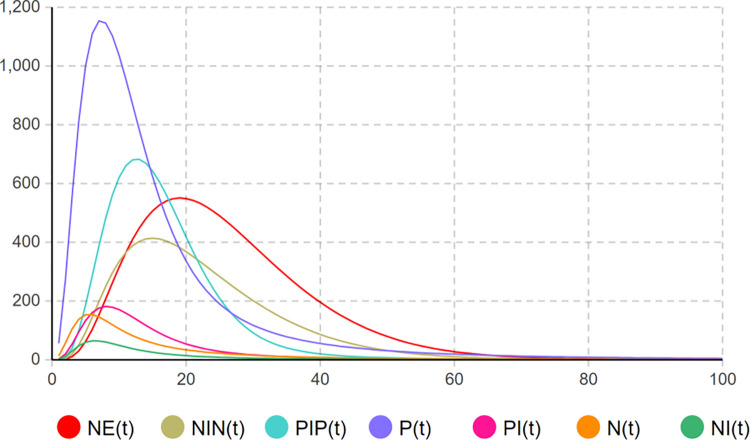
Evolution curve of network public opinion GP and network user traffic in emergencies.

After the system dynamics software is adopted to build the system dynamics model, the set parameter values are input for model simulation, and the user node evolution of the network public opinion group polarization risk simulation model for emergencies is shown in Fig 5.

In Fig 5, the number of users holding a positive opinion reaches a peak at an early stage, and it is higher than the number of users holding a negative opinion. With the development of emergencies, they gradually become users participating in discussions. The curves of *PI(t)* and *NI(t)* are behind the curves of *P(t)* and *N(t)*. Moreover, extreme opinions gradually formed, and the number of users with negative extreme opinions was the largest when t = 20 days. At t = 20 days, although the number of users with negative opinions is small, under the effect of "coupling with other events", the number of users with negative opinions gradually increases. The comparison of the curves of *NE(t)* and *NIN(t)* indicates that not all users have formed extreme opinions, and a considerable number of users have changed to users with negative non-extreme opinions. When t = 20 days, the number of positive opinion users decreases to 1/3 of the peak value, the number of users having a positive opinion and interacting with each other decreases to 1/2 of the peak value, consistent with the phenomenon of "silent spiral" in reality, i.e., users having positive opinions will choose to be silent when they observe that there are more negative opinions in the public opinion field. The extreme opinions account for the mainstream voice position in the network public opinion field when reaching the peak, and the number of users of positive opinions rapidly declines. With the implementation of the life cycle of Internet public opinion information dissemination and the control measures of heat reduction, the extreme opinions decline slowly, and the basic recession ends when t = 60 days. In Fig 3, the search index of "Pfizer COVID-19 small-molecule drug" event rose rapidly around March 17, that is around the time of t = 20 days in the model built in this study. By the end of April, the search index in the model is declining around the time t = 60 days, and the life cycle of network public opinion information dissemination has basically ended. However, around the time of t = 20 days, there were many people with negative emotions in the Internet public opinion field of the "Pfizer COVID-19 small-molecule drug" incident who questioned the entry of Pfizer COVID-19 into the medical insurance and identified the introduction of Pfizer drugs as lacking in patriotism. Thus, the GP simulation model of network public opinion in emergencies built in this study can effectively simulate the real situation of the formation of GP in the event of "Pfizer COVID-19 small-molecule drugs", and it applies to the evolution of GP in different emergency situations. The analysis results can represent the diffusion of extreme opinions under the guidance of the causes and constraints of GP in ternary space. According to the actual situation, sensitivity analysis can be carried out on the parameters of the GP simulation model of network public opinion in emergencies to obtain countermeasures to improve the efficiency of the GP control strategy.

### 4.4 Sensitivity analysis of model parameters

The sensitivity analysis of model parameters includes the analysis of three parameters: the effect of emotional guidance, the effect of heat reduction and the coupling with other events. With other parameters unchanged, the change of model simulation results can be compared and analyzed after a certain parameter is changed.

**Fig 6:**
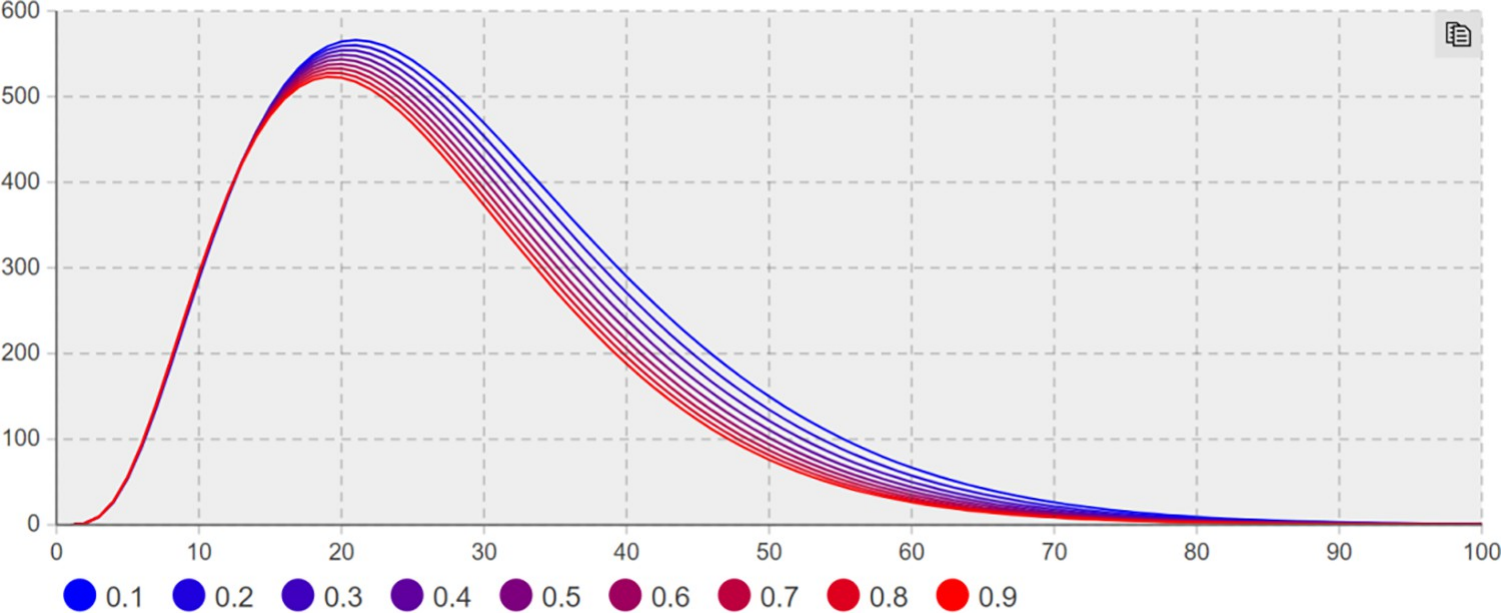
The number of *NE(t)* increases with *λ*_*2*_ change trend.

**Fig 7:**
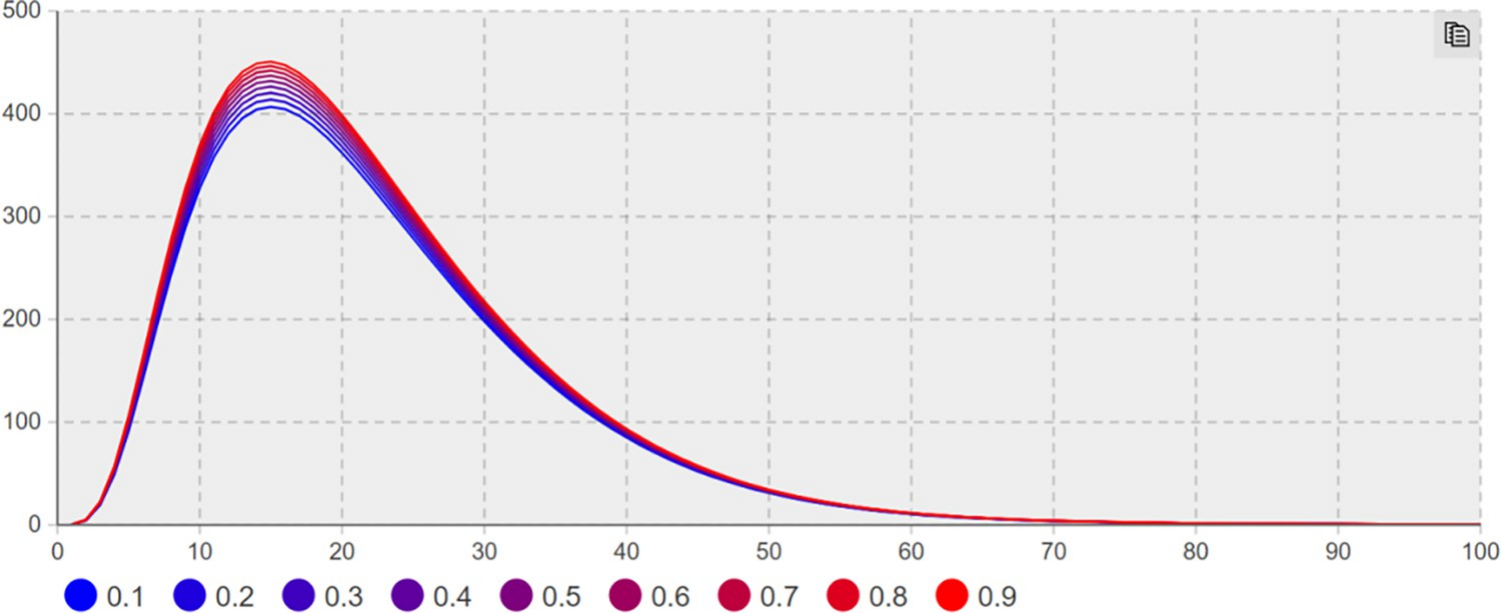
The number of *NIN(t)* increases with *λ*_*2*_ change trend.

(1) Suggesting effect of emotional guidance *λ*_*2*_ sensitivity analysis. Sensitivity analysis is conducted to keep other parameters unchanged. The minimum and maximum values of*λ*_*2*_ are taken as 0.1 and 0.9, respectively, with the step size of 0.1. Figs 6 and 7 presents the trend of the number of network users with negative extreme opinions *NE(t)* and negative non-extreme opinions *NIN(t)* with time based on different effect values of emotional guidance.

In Fig 7 the number of negative non-extreme opinions increased with the increase of the value of *λ*_*2*_. When *λ*_*2*_ *= 0*.*1*, the number of negative non-extreme opinions is 406; when *λ*_*2*_ = 0.9, the number of negative non-extreme opinions reaches 450. In Fig 6, the number of negative extreme opinions varies with the *λ*_*2*_, and the overall change range is similar to Fig 7. As depicted in Figs 6 and 7, under the effect of emotional guidance *λ*_*2*_ from 0.1 to 0.9, the number of extreme negative opinions does not change significantly, consistent with the research content of existing literature [48]. The network public opinion system is a process system rather than an object system, and the dynamic mechanism is capable of changing at different stages through network public opinion propagation. The public opinion exhibits stable randomness in the initial period, non-stationary pseudo-randomness in the outbreak period, as well as non-stationary & non-randomness in the recession period. Accordingly, the effect of emotional guidance on the control of online public opinion is not suggesting significant. In fact, the report on the use of Pfizer drugs in early April 2022 did not significantly affect the negative emotions in the online public opinion field.

**Fig 8:**
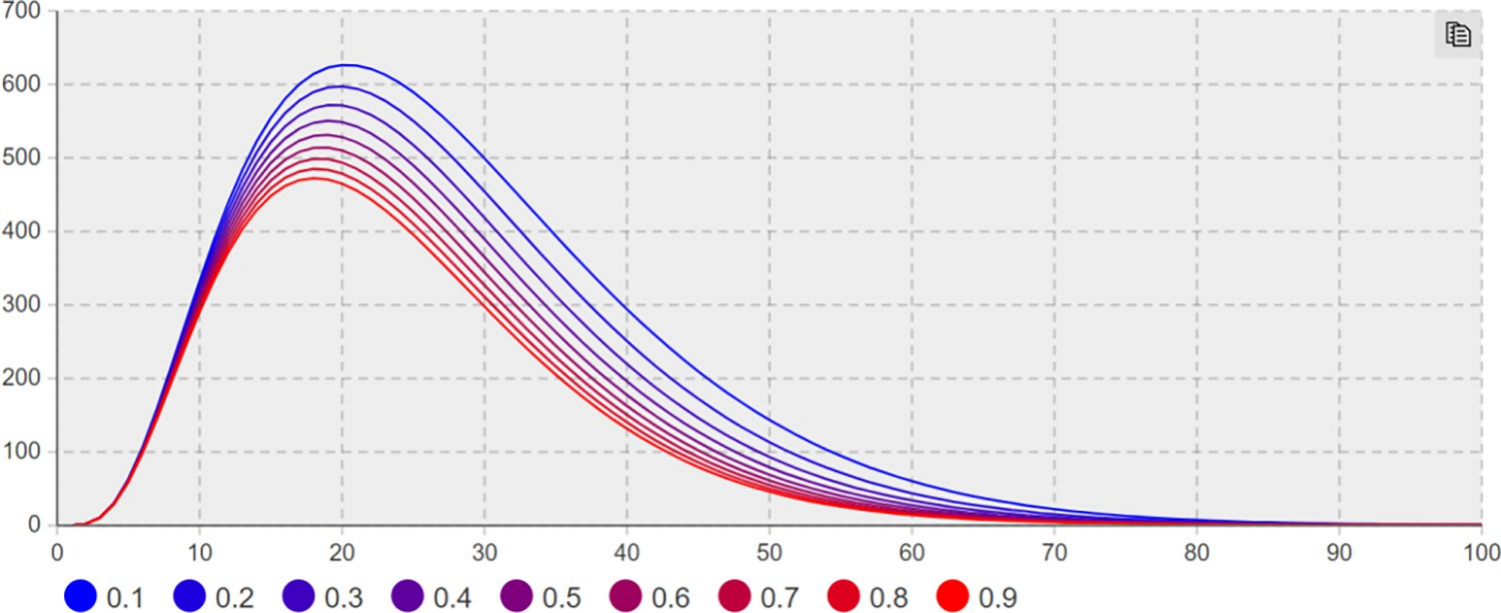
The number of *NE(t)* increases with *λ*_*3*_ change trend.

**Fig 9:**
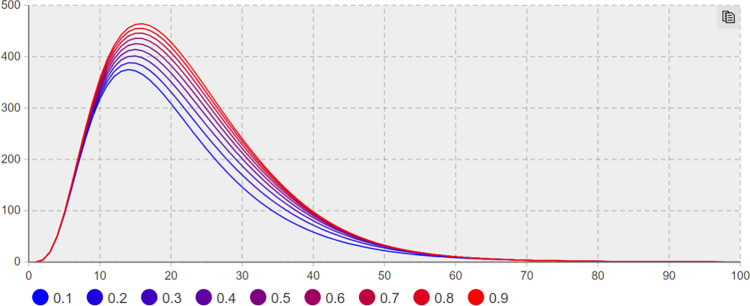
The number of *NIN(t)* increases with *λ*_*3*_ change trend.

(2) Suggesting effect of heat reduction *λ*_*3*_ sensitivity analysis. For the effect of heat reduction *λ*_*3*_, Sensitivity analysis is conducted to keep other parameters unchanged; the minimum and maximum values of *λ*_*3*_ are taken as 0.1 and 0.9, respectively, with a step size of 0.1. Figs 8 and 9 illustrate the trend of the users number with negative extreme opinions *NE(t)* and negative non-extreme opinions *NIN(t)* with time under different heat reduction effect values.

In Fig 9, when*λ*_*3*_ = 0.1 and t = 14 days, the number of network users holding negative non-extreme opinions of *NIN(t)* reaches a maximum of 374. When *λ*_*3*_ = 0.9 and t = 16 days, the number of NIN(t) network users with negative non-extreme opinions reaches a maximum of 464. In Fig 8, when *λ*_*3*_ = 0.1 and t = 20 days, the number of *NE(t)* network users having negative extreme opinions reaches a maximum of 626; when *λ*_*3*_ = 0.9 and t = 18 days, the number of *NE(t)* network users with negative extreme opinions reaches a maximum of 472, and the number of network users with negative extreme opinions decreases by 24.6%. When*λ*_*3*_ = 0.1 and t = 39 days, the number of users with extreme opinions decreases by 50% from the maximum value. When *λ*_*3*_ = 0.9 and t = 33 days, the number of users having extreme opinions declines by 50% from the maximum value, compared with *λ*_*3*_ = 0.1, 6 days in advance. As depicted in Figs 8 and 9, the use of network public opinion control measures to limit the flow and other heat reduction suggesting suppresses extreme opinions significantly.

**Fig 10:**
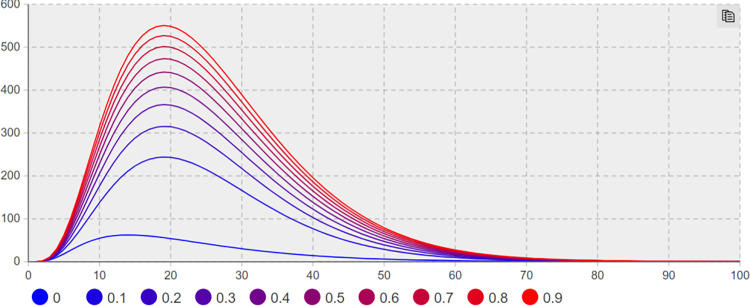
The number of *NE(t)* increases with *λ*_*4*_ change trend.

**Fig 11:**
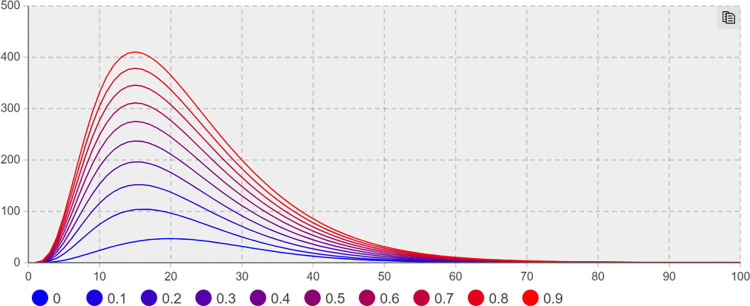
The number of *NIN(t)* increases with *λ*_*4*_ change trend.

(3) Suggesting effect of coupling with other events *λ*_*4*_ sensitivity analysis. Sensitivity analysis is conducted to keep other parameters unchanged; the minimum and maximum values of*λ*_*4*_ are taken as 0 and 0.9, with the step size of 0.1. Figs 10 and 11 present the trend of the number of network users with negative extreme opinions *NE(t)* and negative non-extreme opinions *NIN(t)* with time under different values, respectively.

As depicted in Figs 10 and 11, *λ*_*4*_ significantly affects the number of network users of negative extreme opinion NE(t) and negative non-extreme opinion NIN(t). As depicted in Fig 11, when *λ*_*4*_ = 0 and t = 18 days, the number of network users holding negative non-extreme opinions of NIN(t) reaches a maximum of 46; when*λ*_*4*_ = 0.9 and t = 16 days, the number of network users holding negative non-extreme opinions of NIN(t) reaches a maximum value of 407. Compared with the minimum value of *λ*_*4*_, the difference reaches 88.7%. As depicted in Fig 10, when *λ*_*4*_ = 0 and t = 13 days, the number of *NE(t)* network users with negative extreme opinions reaches a maximum value of 62; when *λ*_*4*_ = 0.9 and t = 19 days, the number of *NE(t)* network users with negative extreme opinions reaches a maximum value of 550. Compared with the minimum value of *λ*_*4*_, the difference reaches 88.73%. When*λ*_*4*_ = 0, the number of extreme opinions users decreases by 50% from the maximum value when t = 29 days. When*λ*_*4*_ = 0.9, the number of extreme opinion users decreases by 50% from the maximum value when t = 37 days, 8 days in advance compared with*λ*_*3*_ = 0.1. Accordingly, if the network users do not have the stereotyped prejudice caused by other events in the sudden crisis, there will be suggesting fewer negative emotions and negative opinions in the network public opinion field significantly, and the extreme opinions can be basically ignored.

## 5. Discussion

### 5.1 Suggesting effect of emotional guidance on GP

After the occurrence of an emergency, the negative emotions such as fear, anger and worry of network users are more likely to spread and even amplify in the network public opinion field. Thus, it is particularly important for the government to pay attention to the negative emotions in the public opinion field under the sudden crisis. The emotional guidance of emergency public opinion mainly includes emergency crisis response in physical space, information disclosure and agenda setting in information space. In this study, according to the actual situation of the "Pfizer COVID-19 small-molecule drug" event, the government’s response impact is placed at the forefront of the model operation, controlling the overall number of users entering the online public opinion field, and indirectly reducing the spread of negative emotions to a certain extent. Information disclosure is also an important means of emotional guidance. The high uncertainty of unexpected events is the main source of negative emotion of network users. The direct result of information disclosure is to reduce the uncertainty of information, and the indirect result is to effectively control the negative emotion in the public opinion field. In this study, the information uncertainty is taken as the parameter of the model operation, and the sensitivity analysis is not made due to the space limitation. In this study, the influence parameter of emotional guidance in the model is*λ*_*2*,_ specific measures such as popular science or other topic settings published by government or media platforms for specific negative emotions. According to the actual situation of the "Pfizer COVID-19 small-molecule drug" event, there is no popular science information released by the official, mainstream media and opinion leaders in the life cycle of public opinion communication. However, news reports on the use of relevant drugs appeared late, and negative and extreme opinions have been established when the ninth edition of the diagnosis and treatment plan formulated by China in 2022. Accordingly, the emotional guidance do not play a significant leading role in the network public opinion field, and there is even an opinion of criticizing the news report hosts. In brief, although emotional guidance serves as a vital strategy for online public opinion control, it should grasp the implementation opportunity and conducted at an accurate time point.

### 5.2 Suggesting effect of heat reduction on GP

Reducing the popularity by restricting the flow and other means has been confirmed as one of the frequently used online public opinion control strategies. The operation mechanism of this strategy is consistent with the dynamic mechanism of network public opinion transmission, and it has been verified in existing research [48]. The heat reduction control strategy for negative extreme opinions in sudden crises has high performance, and it is capable maintaining the information order in information space, blocking the negative feedback of GP on physical space emergencies, and ensuring the formulation of scientific emergency decisions and the implementation of emergency measures. According to the GP simulation model of public opinion in emergencies in this study, the degree of heat reduction could effectively suppress extreme opinions or negative emotions, significantly reduce the number of extreme opinions users, and expedite the cycle of extreme opinions decline. In this study, since the control strategy of heat reduction generally is created and implemented after the formation of extreme opinions, so that it only works during the transition from negative to extreme opinions *NE(t)* to negative to non-extreme opinions *NIN(t)*. In brief, the public opinion control strategy for reducing heat generally occurs after extreme opinions appear, and can achieve good control effect.

### 5.3 Suggesting effect of coupling with other events on GP

The stereotyped prejudice of network users in emergencies originates from other events (e.g., life pressure and improper government emergency management). The life pressure of sudden natural disasters, sudden accidents and social security incidents on the masses is generally limited to the space where the emergencies occur, and will not affect the daily life of considerable people. However, public health emergencies involve a large space. Notably, the daily life of the people in a certain area will be affected to varying degrees under health emergencies. The fear and impatience arising from life pressure or improper government emergency management will be directly mapped into information space. When the number of network users with similar situations reaches a certain number, negative emotions will spread rapidly while easily generating extreme opinions. Stereotyped prejudice is capable of accelerating the formation and polarization of extreme opinions, while making network users who originally hold positive opinions tend to have negative opinions and then have extreme opinions. According to the simulation results of this model, the coupling with other events has a significant impact on the negative extreme opinion *NE(t)* and the negative non-extreme opinion *NIN(t)*. The above analysis suggests that ensuring people’s livelihood and scientific response in emergencies is an important content of emergency management and takes on a critical significance to disaster relief and urban recovery resilience.

### 5.4 Analysis of fidelity and extrapolation of model

In this study, the GP risk of public opinion in emergencies is evaluated using the simulation method, such that the model simulation reality should exhibit reasonable fidelity and high extrapolation. Fidelity expresses the difference between the model and the real object. The higher the fidelity, the closer it will be to the real object. The concept of fidelity has been usually employed in engineering technology or scientific research to express the close to real characteristics of some mathematical models in simulation. Extrapolation is the universal description of the simulation model applicable to a certain type of scenario. The higher the extrapolation, the stronger the applicability will be in the face of changes in a wide variety of factors in a specific scenario. Fidelity and extrapolation are contradictory indexes that promote each other in simulation model evaluation. In fact, more information is the common guarantee of fidelity and extrapolation. However, a specific event requires a high degree of fidelity, thus inevitably affecting the extrapolation of the simulation model and vice versa.

As mentioned above, the simulation model built in this study fully indicates the mapping of the content in physical space and social space to information space through collaborative perception, as well as the information integration and analysis under the coupling effect of ternary space in information space. Moreover, the introduction of the dynamic mechanism of network public opinion propagation further enhances the fidelity of the simulation model. The comparison between Fig 3 with the empirical research results of this study indicates that GP occurred near the 20th day after the outbreak of public opinion events. In the simulation model built in this study, the suggesting effect of the degree of heat reduction will play a certain role after the formation of the GP, such that the GP enters recession. However, the government did not take considerable important measures to restrict the flow of the "Pfizer COVID-19 small-molecule drug" incident. Thus, Fig 3 suggests that the popularity of public opinion lasts for a relatively long time compared with the simulation results.

## 6. Conclusion

The main challenge of this article is the structuring of GP influencing factors (simulation parameters). It is necessary to summarize typical and independent simulation model parameters among numerous influencing factors. We proposed the structural framework of GP risk of public opinion of emergencies in the ternary space, and the causes and constraints of GP distributed in the ternary space are considered. On that basis, the GP risk of public opinion of emergencies is indicated by the number of network users with extreme opinions, and a simulation model of GP is built. The system dynamics simulation of the model is conducted using the software Anylogic, and the evolution trend of the model is predicted from the perspective of simulation. The above results are compared with the real situation of the "Pfizer COVID-19 small-molecule drug" incident, which confirmed the universality of the public opinion GP model. Based on the sensitivity analysis of model parameters, it is found that the public opinion control strategy of heat reduction can achieve good control effect, and coupling with other events has a significant impact on the formation of extreme opinions.

The main theoretical contribution of this study is to analyze the public opinion GP of emergencies from the perspective of ternary space, considering the effect of government response, emotional guidance, heat reduction and coupling with other events in physical space, user risk propensity in social space and the effect of information uncertainty on the formation and dissemination of extreme opinions in information space. The main practical contributions of this study is through the system dynamics simulation method, the GP of public opinion in emergencies could be predicted using the model, and the universality of the model is determined through the empirical analysis, thus complementing the research method of GP in the public opinion field. The practical significance of this study is to focus on the GP of emergency network users and the formation and dissemination of negative emotions and extreme opinions. It is proposed to grasp the opportunity of emotional guidance, adopt the control strategy of reducing the heat of public opinion, and ensure the people’s livelihood and scientific response under emergency crises.

The limitation of this study lies in the insufficient explanation of the negative effect of GP on emergencies. The negative opinion in the GP often has an adverse impact on the decision-making in the emergency management, aggravating the crisis of emergencies, and thus forming a vicious circle of emergency crisis in the ternary space. This process is a complex system of continuous iteration, and the model built in this study lacks the revelation of this iterative process. In the future, we will continue to study the negative effect of GP on emergencies in physical space.

## Supporting information

S1 Fig (TIF)

S2 Fig (TIF)

S3 Fig (TIF)

S4 Fig (TIF)

S5 Fig (TIF)

S6 Fig (TIF)

S7 Fig (TIF)

S8 Fig (TIF)

S1 Table (DOCX)

S2 Table (DOCX)

S1 File Model. (ALP)
