# Supplementary figures and images for: Research on risk assessment model and simulation of online group polarization in emergencies
Source: PLoS One. 2024 Jun 17;19(6):e0305552. doi: 10.1371/journal.pone.0305552 (PMC11182558; doi:10.1371/journal.pone.0305552)

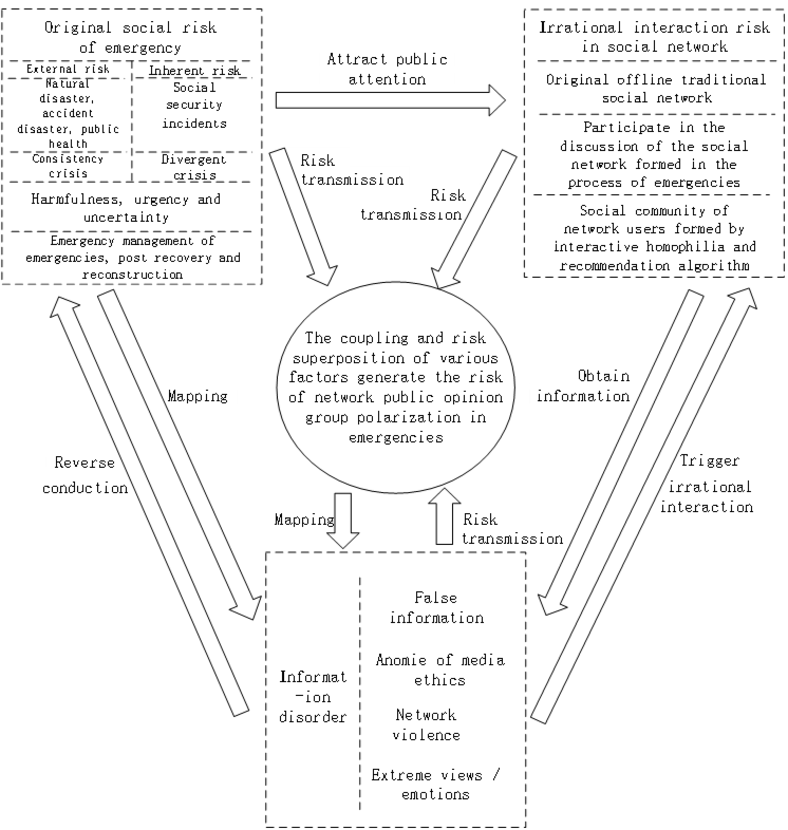

Supplement: S1 Fig — (TIF) [file pone.0305552.s001.tif]

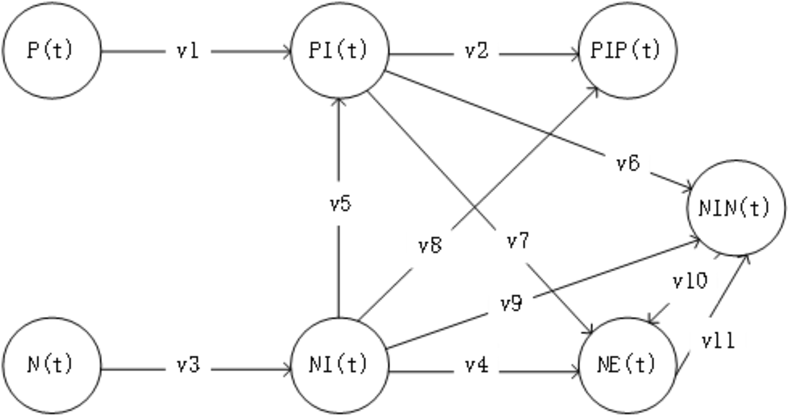

Supplement: S2 Fig — (TIF) [file pone.0305552.s002.tif]

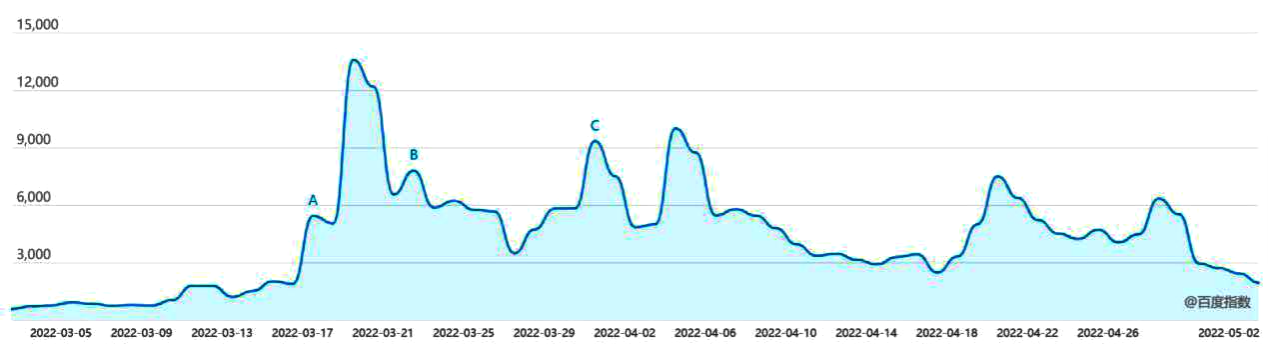

Supplement: S3 Fig — (TIF) [file pone.0305552.s003.tif]

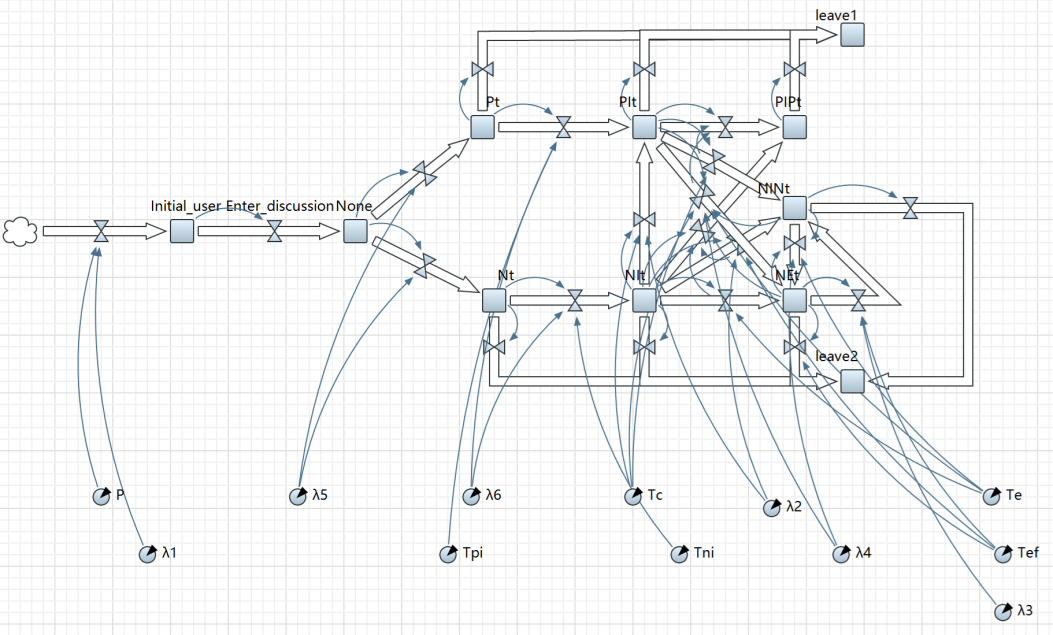

Supplement: S4 Fig — (TIF) [file pone.0305552.s004.tif]

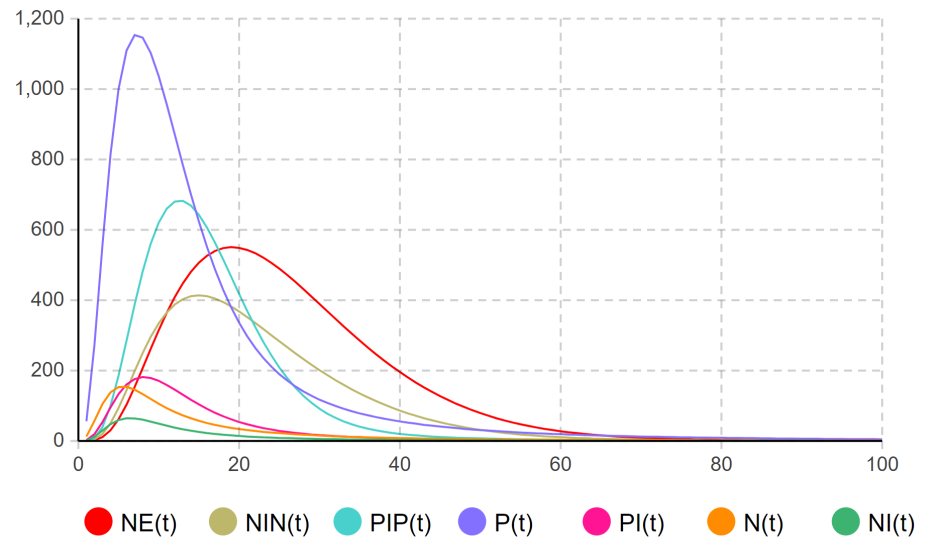

Supplement: S5 Fig — (TIF) [file pone.0305552.s005.tif]

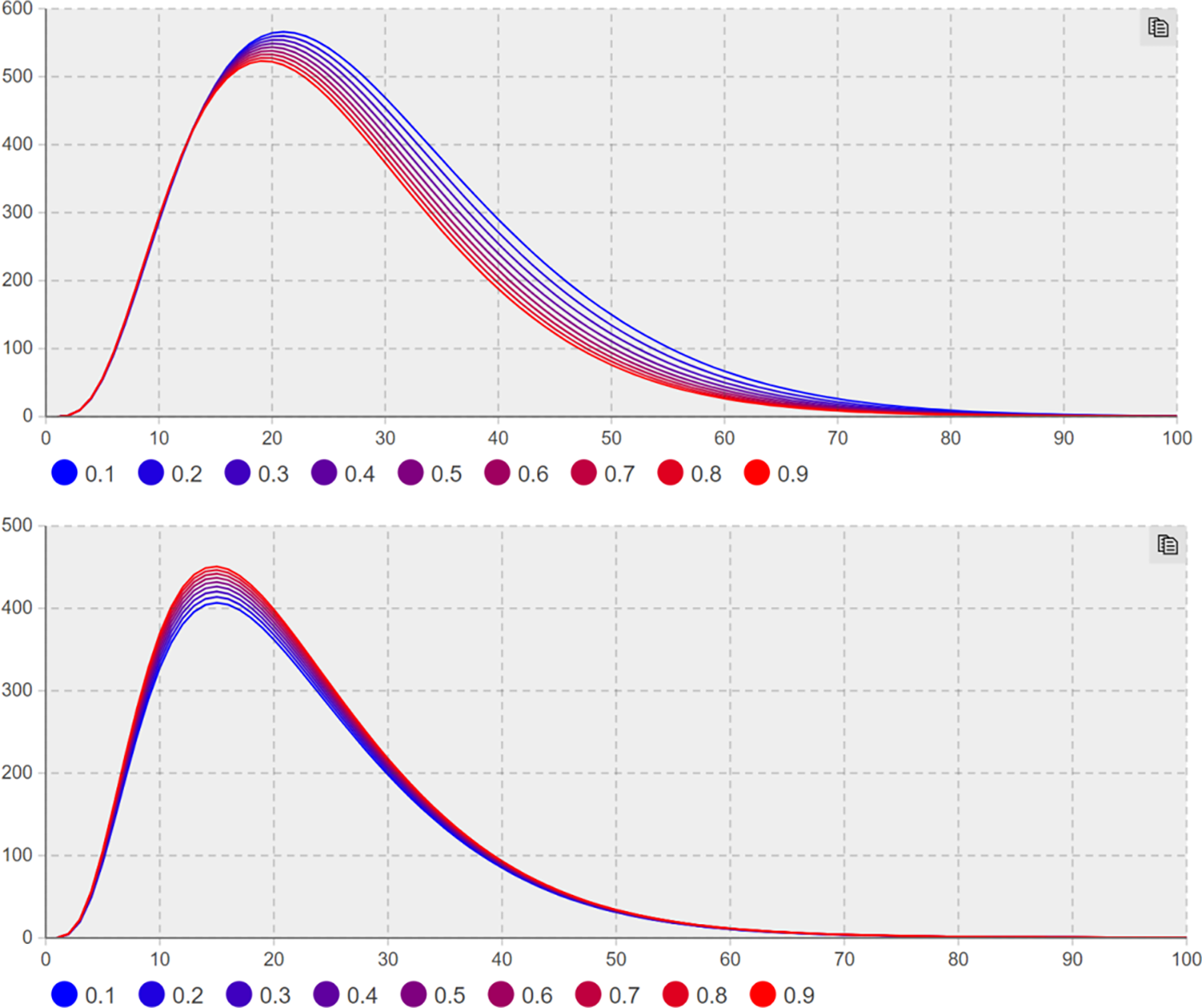

Supplement: S6 Fig — (TIF) [file pone.0305552.s006.tif]

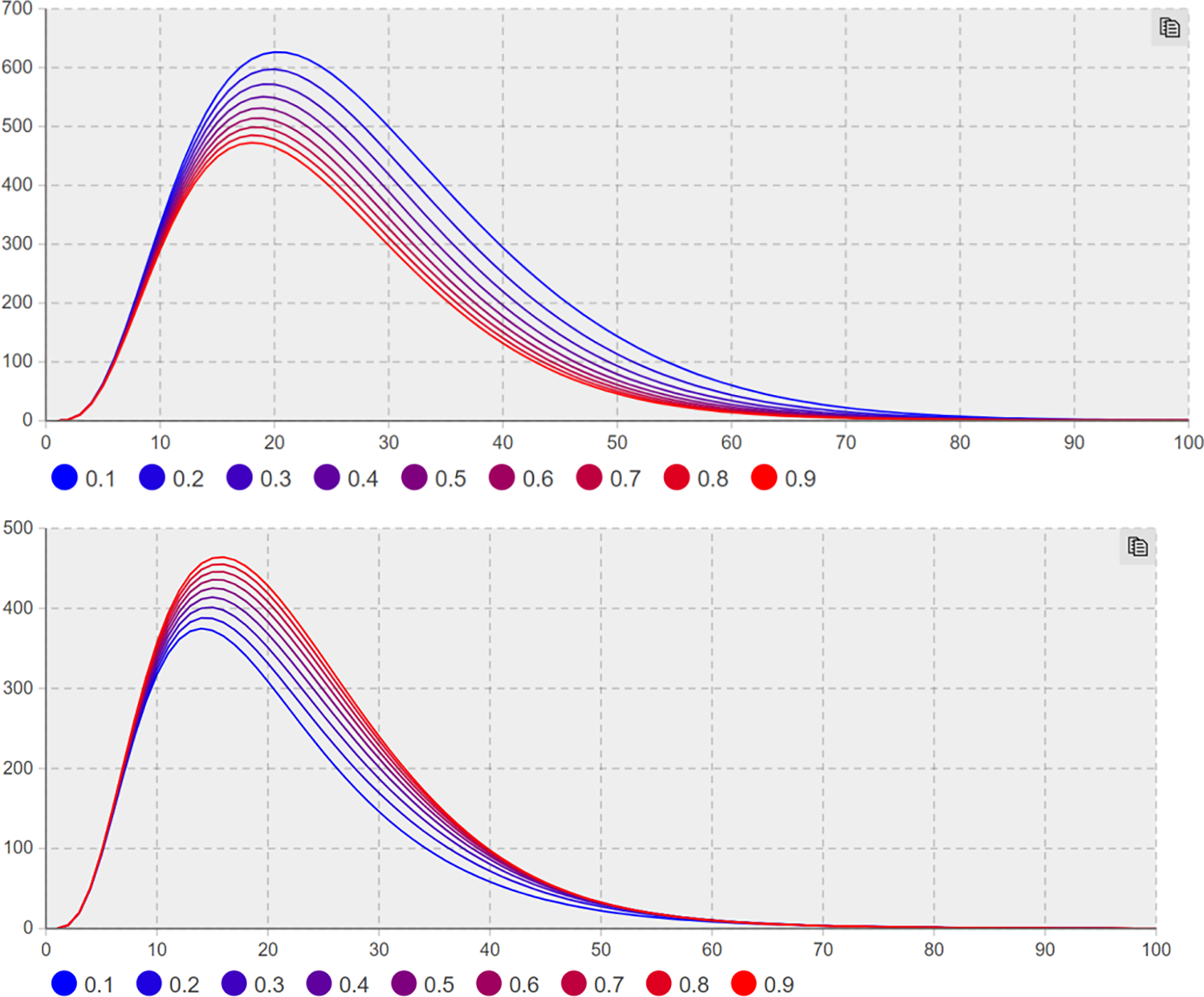

Supplement: S7 Fig — (TIF) [file pone.0305552.s007.tif]

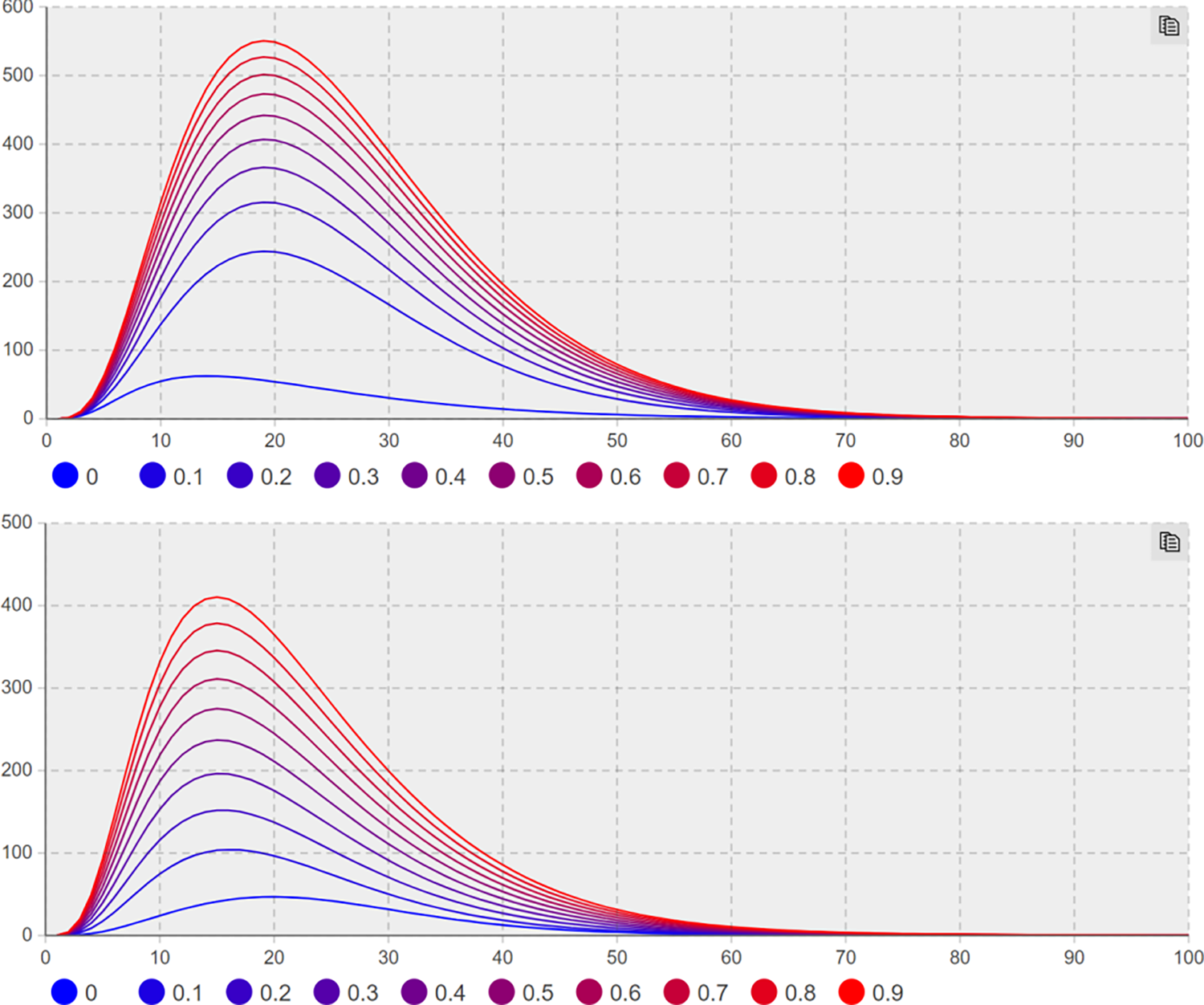

Supplement: S8 Fig — (TIF) [file pone.0305552.s008.tif]
