# Supplementary material for: Research on risk assessment model and simulation of online group polarization in emergencies
Source: PLoS One. 2024 Jun 17;19(6):e0305552. doi: 10.1371/journal.pone.0305552 (PMC11182558; doi:10.1371/journal.pone.0305552)
Supplement: S1 Table — (DOCX) [file pone.0305552.s009.docx]

**Table 1. Interpretation of relevant indicators of GP risk assessment model of network public opinion in emergencies**

| **Parameter** | **Parameter meaning** | **Parameter** | **Parameter meaning** |
| --- | --- | --- | --- |
| **t** | At a certain time in the process of network public opinion propagation of emergencies t | ***V6*** | Change rate of users holding positive opinions to users holding negative non-extreme opinions |
| ***P(t)*** | At a certain time t, the number of users with positive opinions at the beginning of network public opinion focusing on emergencies | ***V7*** | Change rate of users holding positive opinions to those holding negative extreme opinions |
| ***PI(t)*** | The number of users who in P(t) interact with other users at a certain time t | ***V8*** | Change rate of users with negative opinions changing to positive opinions after participating in the discussion |
| ***PIP(t)*** | At a certain time t, the number of users with positive opinions after interacting with other users | ***V9*** | Change rate of users holding negative opinions to users holding negative non-extreme opinions |
| ***N(t)*** | At a certain time t, the number of users with negative opinions at the beginning of network public opinion focusing on emergencies | ***V10*** | Change rate of users from negative non-extreme opinions to negative extreme opinions |
| ***NI(t)*** | Number of users who in N(t) interact with other users at a certain time t | ***V11*** | Change rate of users from negative extreme opinions to negative non-extreme opinions |
| ***NE(t)*** | At a certain time t, the number of users with extreme opinions with negative emotions | ***λ_1_*** | Government response impact |
| ***NIN(t)*** | At a certain time t, the number of users with negative emotions and non-extreme opinions after interacting with other users | ***λ_2_*** | Effect of emotional guidance |
| ***V1*** | Change rate of user participation in discussions with positive opinions | ***λ_3_*** | Effect of heat reduction |
| ***V2*** | Users having positive opinions maintain the change rate of positive opinions after participating in the discussion | ***λ_4_*** | Coupling effect of other events |
| ***V3*** | Change rate of users with negative opinions participating in discussions | ***λ_5_*** | User risk propensity |
| **V4** | Users with positive opinions will generate negative extreme opinions after participating in the discussion | ***λ_6_*** | Information uncertainty |
| **V5** | Change rate of users holding negative opinions to users holding positive opinions | - |  |
