# Supplementary material for: Research on risk assessment model and simulation of online group polarization in emergencies
Source: PLoS One. 2024 Jun 17;19(6):e0305552. doi: 10.1371/journal.pone.0305552 (PMC11182558; doi:10.1371/journal.pone.0305552)
Supplement: S2 Table — (DOCX) [file pone.0305552.s010.docx]

**Table 2. Parameter setting of GP simulation model of network public opinion in emergencies**

| **parameter** | **Value meaning of parameter value setting** | **Parameter value** |
| --- | --- | --- |
| **S（0）** | Number of initial users in the emergency network public opinion field | 100 people |
| ***λ_1_*** | The effect of government response measures on the number of users who pay attention to Internet public opinion events under emergency crises | 0.2 |
| ***λ_2_*** | The effect of the emotional guidance measures taken by the management department against the negative opinions in the public opinion field on the attitude change of network users | 0.2 |
| ***λ_3_*** | The effect of traffic restriction measures taken by the management department against extreme opinions in the public opinion field on the attitude change of network users | 0.4 |
| ***λ_4_*** | Under the sudden crisis, coupling with other events, the network users have a negative impact on the users or changes to a negative opinion | 0.9 |
| ***λ_5_*** | The proportion of Internet users with negative emotion tendency due to their individual personality characteristics | 0.2 |
| ***λ_6_*** | The effect of network users' interactive discussions with other users due to the uncertainty of information of unexpected events | 0.5 |
| ***T_c_*** | Time required for positive and negative opinions to change each other | 2 days |
| ***T_e_*** | Time required to form extreme opinions | 6 days |
| ***T_ef_*** | Time required for extreme opinions to subside | 7 days |
| ***T_pi_*** | Interaction time between network users with positive opinions and other users | 3 days |
| ***T_ni_*** | Interaction time between network users with negative opinions and other users | 1 days |
